# Visualizing contextual determinants in and across heterogeneous settings: a qualitative study on structured school health promotion implementation

**DOI:** 10.1186/s43058-026-00861-x

**Published:** 2026-01-16

**Authors:** Katharina Sterr, Deborah Cragun, Filip Mess, Friederike Butscher, Monika Singer, Simon Blaschke

**Affiliations:** 1 TUM School of Medicine and Health, Department of Health and Sport Sciences, Technical University of Munich, Am Olympiacampus 11, 80809 Munich, Germany; 2 College of Public Health, Department of Community Health Sciences, University of South Florida, 3720 Spectrum Blvd, Suite 304, Tampa, FL 33612 USA

**Keywords:** School Health Promotion, Capacity-Building, PDSA cycle, Context, Multi-Site Implementation, Cross-Case Comparison, Matrix Heat Map, Data Visualization

## Abstract

**Background:**

Schools have the potential to promote equitable health from early life onwards yet require sufficient organizational capacity to achieve sustained action. Structured improvement approaches, such as PDSA cycles, may help strengthen this capacity by guiding systematic implementation processes. However, their potential in school health promotion remains insufficiently understood, particularly regarding the heterogeneous contextual factors shaping their application. This study examined which contextual determinants shape schools’ perceived implementability of the PDSA cycle for health promotion and how these conditions differ across schools.

**Methods:**

Nine German primary schools participating in a holistic health promotion program were purposively sampled to capture heterogeneity across federal states, socioeconomic contexts, and urban–rural settings. Semi-structured qualitative group interviews in a workshop format were conducted with school principals, teachers, and parents and analyzed using the framework method guided by the CFIR. To facilitate cross-case comparison, color-coded valence ratings (facilitator/barrier/mixed) were visualized in a Matrix Heat Map, enabling identification of contextual tendencies.

**Results:**

Fifteen contextual factors emerged across the CFIR domains of Outer Setting, Inner Setting, and Individual. Schools with prior experience using structured processes similar to PDSA cycles reported more facilitators, such as established communication structures, while schools without such experience perceived more barriers, notably financial constraints. Common barriers across schools included limited parental engagement and staff shortages, whereas leadership support and compatibility of program components were consistent facilitators. Some factors interacted dynamically, with resource constraints reinforcing other barriers or with strong mission alignment amplifying engagement.

**Conclusion:**

Schools’ prior structured experience seemed to be associated with how they perceived the implementability of PDSA cycles for health promotion implementation, with more experienced schools anticipating more facilitators and fewer barriers. While causality cannot be inferred, these exploratory findings are hypothesis-generating and suggest that prior structured experience may be an important factor to consider for tailoring implementation support and building organizational capacity. Beyond these insights, extending the framework method with a color-coded Matrix Heat Map proved valuable for visualizing contextual heterogeneity and revealing tendencies across cases. This combined approach may inspire further research on how contextual configurations shape the use of structured processes in complex, multi-site implementation settings.

**Supplementary Information:**

The online version contains supplementary material available at 10.1186/s43058-026-00861-x.

Contributions to the literature

- Structured approaches such as the PDSA cycle are increasingly promoted to build capacity for school health promotion, yet their implementability remains insufficiently understood and may both shape and be shaped by contextual factors.
- This study qualitatively explores such contextual determinants across multiple schools by combining the framework method with a visualization for cross-case comparison, illustrating a transparent and transferable way to capture contextual heterogeneity of implementation settings.
- By identifying a cross-case tendency related to prior structured experience alongside other key factors, the findings are hypothesis-generating and provide a foundation for future implementation research.

## Background

Transforming the environments where we live, learn, and work into health promoting settings has been a central goal for health professionals since the World Health Organization's (WHO) *Ottawa Charter* was published in 1986 [1].Given that good health and healthy behaviors early in life are linked to better health throughout the lifespan, focusing on the settings where children live and learn is crucial from a health promotion perspective [2, 3]. Schools offer a key opportunity for impactful health promotion and the advancement of health equity, as they are established and accessible platforms providing the structures to engage with a great number of children, regardless of socioeconomic factors or cultural and political background [4–6].

Research shows that holistic school health promotion, following the Health Promoting School framework proposed by the WHO, can bring about sustainable and equitable health outcomes [7–9]. This framework provides guidance for the implementation of interventions targeting individual determinants by focusing primarily on the creation of a healthy school environment that enables the school community to engage in health-promoting behaviors and strengthen health-promoting community connections outside of school [10].Today, a health promoting school, is characterized as a school “that constantly strengthens its capacity as a healthy setting for living, learning and working” [11]. Building capacity refers to the process of developing and enhancing the abilities, resources, and structures necessary for schools to effectively implement and sustain health promoting activities (HPA) [12].

Despite promising evidence that health promoting schools can improve health and educational outcomes and the ongoing call by the WHO to “make every school a health-promoting school” [7–9, 13], the landscape remains fragmented. Many schools implement intervention after intervention without strengthening organizational capacity, resulting in “projectitis” and straining human and financial resources [4, 14–16]. Accordingly, there is a strong need to support schools in building organizational capacity for health promotion [4, 17].

One promising way to strengthen this capacity may be to provide schools with tools to design and manage change processes effectively [4, 18–20]. The Plan-Do-Study-Act (PDSA) cycle, widely used in quality improvement initiatives, is one of the most established strategies for guiding structured change processes in organizations [21]. Beyond supporting individual projects, it has been described as a mechanism for organizational learning and capacity building, enabling teams to adapt and sustain improvements over time [21, 22]. In the school context, PDSA-like improvement cycles have similarly been highlighted as a way to strengthen systems that support implementation, thereby contributing to the development of organizational capacity for change [23]. This rationale has been taken up in practical recommendations, for example, the Schools for Health in Europe Network Foundation (SHE) recommends cyclical processes such as the PDSA cycle to become a health promoting school [20, 24], and in Germany, the national Guideline for Prevention and Health Promotion also advises integrating the PDSA cycle into setting-based interventions to foster capacity building and sustainability [25].

At the same time, research shows that PDSA cycles are often applied inconsistently or superficially, which can limit their potential to build capacity [22]. Moreover, contextual factors such as leadership support, work infrastructure, and time play crucial roles in both school improvement and school health promotion implementation implicating that these factors might also shape how schools can apply the PDSA cycle for implementing HPA [23, 26–29]. Contextual factors, often categorized using the Consolidated Framework for Implementation Research (CFIR; [30, 31] can both facilitate and hinder implementation and sustainment and schools seem to differ substantially in these conditions, which underscores the heterogeneity of schools and complexity of implementing health promotion in educational settings [27, 28, 32]. To account for this, and, with a view to improving transferability, it is essential to explore which contextual factors may be relevant across schools and which differ from school to school, when it comes to PDSA cycle utilization. Therefore, before evaluating whether the PDSA cycle is an effective strategy for implementing HPA, we first aim to understand these core contextual factors and how they may shape PDSA utilization.

Methodologically, this requires an approach that allows for in-depth qualitative exploration while also enabling comparison across multiple cases without losing contextual richness. Building on earlier work that used a color-coded Matrix Heat Map (MHM) to visualize contextual determinants in healthcare organizations [33], we combined the framework method [34], guided by the CFIR, with a similar visual extension.

Against this backdrop, the aims of this paper are exploratory in nature. Specifically, we seek (1) to identify the contextual factors perceived as key determinants (i.e., barriers or facilitators) for the utilization of the PDSA cycle in implementing HPA, (2) to examine which of these factors differ across schools, (3) to explore whether such differences reveal notable tendencies across cases, and (4) to reflect on the added value of extending the framework method with a MHM for implementation research.

## Methods

### Study setting

Data for this study were collected in December 2023 and January 2024 as part of the implementation evaluation of a holistic school health promotion program in German primary schools (three cohorts with 300 schools each). Schools from all over Germany were recruited via email, following a detailed sampling strategy to achieve maximum heterogeneity and include at least 25% of schools from socio-economically disadvantaged areas [35] (Additional File 1). The program encompasses individual-level and organizational-level HPA as well as capacity-building components. An example of individual HPA is the provision of teaching materials on nutrition to be used by the teachers in class. An example of an organizational HPA is the provision of equipment for physical activity to be handed out to the pupils. However, capacity-building is the program’s primary focus. Schools are trained to apply a structured PDSA cycle for their HPA (that they select), now and in the future. At the start of the program, a so-called steering group has to be established at each school, involving the principal, two teachers and a parent representative. Their task is to lead the implementation and change process. Throughout this process, schools are supported through implementation strategies such as trainings, counseling with individual contact persons, get-together meetings, and materials. After two years, the aim is that schools are able to conduct HPA on their own following the PDSA cycle in a structured way. All participating schools are offered the same program content and implementation support, regardless of individual school context. Ethics approval was obtained for all data collection procedures of this evaluation project from the ethical committee of the Technical University of Munich (2023–12-NM-KH).

### Positionality, reflexivity and rigorous reporting

The lead researcher, KS, a 28-year-old woman who grew up and went to school in Germany, has prior experience in qualitative research, particularly the framework method [34] and thematic analysis [36]. Other researchers involved in data collection or analysis (SB, MS, DC) also have qualitative research experience. We adopted a critical realist ontology, aligned with our research goals and methodology, and contextualism as our epistemology, assuming an objective reality that is perceived and constructed contextually [37]. Further, we engaged in reflexive practices throughout the research process. Team members brought complementary expertise from public health (setting-based health promotion such as school, urban and workplace health), education, and implementation science, which facilitated critical discussion of potential biases and blind spots. Case knowledge gained during fieldwork, such as impressions of schools and participants’ modes of self-presentation, was explicitly acknowledged as an interpretative resource rather than a limitation. Debriefings among authors provided the opportunity to negotiate divergent perspectives, and to strengthen shared understanding of which contextual factors were most salient in the data. In this way, subjectivity was systematically harnessed as a source of analytical depth rather than treated as a weakness. To ensure rigorous reporting, we follow the standards for reporting qualitative research (SRQR) (Additional File 2) [38].

### Sampling

We selected primary schools from cohort two (intervention on-boarding in late fall 2023) following the purposeful maximum heterogeneity sampling approach by Patton et al. in order to represent the cohort as best as possible [39]. Factors considered for sampling were urban or rural area, socio-economic index and federal state, as the educational systems slightly differ between the federal states in Germany. The aim was to include one school per federal state. A sample was drawn, which resulted in N = 16 schools, 50% of them located in urban areas, and 50% from lower socio-economic index areas. The sampled schools were contacted via email and informed about the planned study procedure. Of the 16 invited schools, nine agreed to participate; reasons for non-participation are reported in the results section. After members of the steering group (teachers, principals and parent representative) of the respective school agreed to participate and sent their signed participant information sheets, an appointment was scheduled for either online or on-site data collection, depending on the school's preference.

### Data collection and analysis

To obtain in-depth insights without creating the impression of evaluation or surveillance, we conducted qualitative semi-structured group interviews in a workshop format [40]. While sharing features with focus groups, we deemed the label less suitable because some groups included participants across hierarchical levels (e.g., principals and teachers) and we incorporated participatory mapping exercises. Such adaptations move beyond the classic focus group definition [41] and align more closely with workshop methodologies that foster joint meaning-making [42]. We therefore describe our approach as a workshop format, positioning participants as experts and ourselves as learners to underscore the value of their perspectives. To ensure comparability with existing literature, the interview guide was based on the CFIR [30, 31], widely used to categorize implementation determinants as barriers and facilitators. The framework comprises five domains (Innovation, Inner Setting, Outer Setting, Individual, and Process) with a total of 39 constructs as well as implementation outcomes [43]. In consultation with the research team, we selected relevant constructs and excluded the Innovation Domain (as it pertains to the intervention itself) [44] and the Process Domain (as data collection occurred at the program’s onset). For the same reason, *implementability* was used when eliciting possible barriers and facilitators to the utilization of the PDSA cycle. Damschroder et al. define *implementability* as “the likelihood the innovation will be put in place or delivered”, thus a forward-looking concept [43]. Interviewees were therefore asked to name barriers and facilitators that they think will impact the successful utilization of the PDSA cycle to HPA implementation in the future. Given that prior experience with PDSA cycle-like processes may be an important determinant of successful implementation of future HPA [26], we also asked interviewees about the extent to which the implementation of HPA has already been approached using a structured process, similar to the PDSA cycle. The workshop format was set up to work both on-site as well as online, was conducted by two researchers (always KS with another person who took field notes) and all interviews were recorded. A detailed description of the workshop format is provided in Additional File 3.

For the management and data analysis procedure, the framework method with its seven steps was chosen as a suitable methodological guideline, aligning with Salvati et al.’s approach and our ontological and epistemological position [33]. The framework method is particularly suitable for the aims of comparing contextual factors across schools and identifying potential patterns, based on qualitative data gathered through interviews [34]. We extended the framework method through color-coding the final framework matrix and using the resulting MHM for further cross-case comparisons. Although iterative, our method included the following steps:

- (1) Transcription & (2) Familiarization with the interviews

All interviews were transcribed verbatim in the same format, pseudonymized, and KS and MS familiarized themselves with the interviews and the field notes.

- (3) Coding

First, the updated CFIR (version 2.0) was used as a codebook to test-code three interviews by hand. During this process, the researchers (KS and MS) adapted it to the school context, took notes about ambiguities in the coding, extended it through inductively derived categories, merged some categories and overall revised the codebook. In a second round, the adapted codebook was tested again with the same three interviews and further adaptations were made until all ambiguities in coding were resolved.

- (4) Developing a working analytical framework & (5) Applying the analytical framework

The codebook, as well as the adaptation steps are documented in Additional File 4. For the final coding, the codebook and all transcripts were uploaded to MAXQDA 2020 [45], a software facilitating qualitative research. All transcripts were double-coded by two researchers (KS and MS) following the codebook, and valences were assigned to coded data indicating whether a contextual factor was perceived as a facilitator (positive), barrier (negative) or as mixed [31, 33]. All conflicts were resolved through discussion.

- (6) Charting data into the framework matrix & creating a MHM

Spreadsheets were created for each school with CFIR domains and constructs on the x-axis and all related quotes imported from MAXQDA 2020 [45]. Thereby, one construct could be represented by one or multiple quotes per interview. Valence ratings were assigned color codes and the cell with the quotation was colored accordingly: facilitators in blue, barriers in red and mixed factors in purple. If, for example, a quote was that *the schoolyard offers a lot of space for the implementation of movement*, this quote would be inserted into the *Inner Setting/Space* column and, as a facilitator, colored blue. A mixed rating, colored purple, could either come from two or more contrasting statements in one construct or one statement (e.g. *some teachers are really motivated, others don’t care at all* in the *Individual Domain/Teachers)*. Next, all valence ratings per construct were aggregated into a final rating based on the following scheme:

- Blue only = blue = positive
- Blue AND purple only (no red) = blue/purple = mixed positive
- Blue AND red (AND purple) = purple = mixed
- Purple only = purple = mixed
- Red AND purple only (no blue) = red/purple = mixed negative
- Red only = red = negative
- Grey = neutral, not mentioned

For prior experience with a structured process such as the PDSA cycle, we initially expected the responses to be categorized similarly to the other factors. However, the responses turned out to be dichotomous, with schools reporting either having or lacking prior experience with the utilization of structured processes like PDSA cycles. Consequently, we used blue to indicate prior experience (present) and red to indicate no prior experience (absent).

This procedure was repeated for each school, generating individual spreadsheets with final valence ratings. Another spreadsheet with constructs on the x-axis and schools on the y-axis was then created, where all ratings were imported and merged into a MHM to facilitate visual cross-case comparison.

- (7) Interpreting the data (cross-case comparison)

Schools were grouped within the MHM based on whether they reported prior experience using structured PDSA-like processes. Both the in-depth insights from the interviews and the visualization in the MHM were evaluated and compared across schools by the principal researcher and DC in order to identify similarities and differences based on whether the schools (i.e., cases) have utilized PDSA cycles.

## Results

### Sample

Of the 16 schools invited, nine agreed to participate, with a total of 20 representatives. Three schools declined due to lack of time and resources (e.g., staff). Some schools also cited limited familiarity with the program as a reason for non-participation, although this was not an exclusion criterion. Four schools did not respond despite two reminders. Table 1 provides an overview of the characteristics of the participating schools.

**Table 1:** School Characteristics

School	Federal State	Urban/Rural^a^	Socio-economic index^b^	# of Students	Interview format	# of Representatives & Roles
A	Berlin	Urban	1	422	Online	2; Principal, School psychologist
B	Bavaria	Urban	1	161	On-site	2; Principal, Teacher
C	Bavaria	Rural	0	205	On-site	2; Teachers
D	Baden-Württemberg	Rural	0	60	On-site	3; Principal, Teachers
E	Saxony-Anhalt	Rural	0	176	Online	1; Teacher
F	Brandenburg	Rural	1	450	Online	3; Principal, Parent, Teacher
G	Rhineland-Palatinate	Urban	0	68	Online	2; Principal, Teacher
H	Saarland	Urban	0	191	Online	2; Teacher
I	Hesse	Urban	1	380	On-site	3; Teacher, Parents

Schools provided information on federal state and number of students upon registration for the program

^a^Based on data by the Federal Institute on Building, Urban Affairs and Spatial Development, 2021

^b^Based on data by the SINUS Market and Social Research Institute, 2022

### Contextual factors across schools

**Fig. 1:**
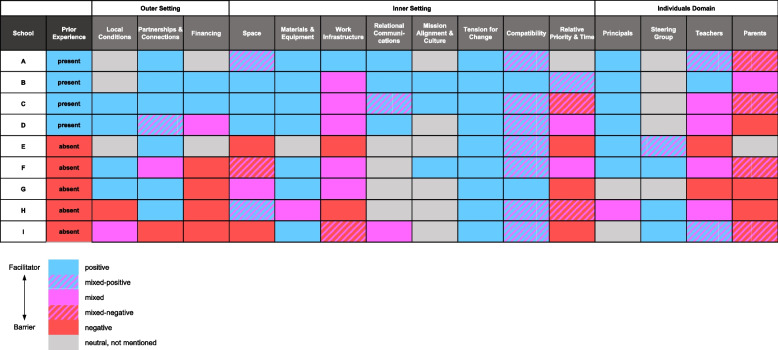
Matrix Heat Map The MHM shows each individual school in a row, ordered from a) whether they reported prior experience (present at the top, absent at the bottom) and b) from schools reporting fewer barriers (top) to schools reporting most barriers (bottom). Each column represents a contextual factor, with colors representing the valence rating

In the interviews, 15 contextual factors were mentioned, spanning the CFIR domains of the Outer Setting, Inner Setting, and Individual. The MHM (Fig. 1) reveals commonalities and differences in how contextual factors were perceived across the four schools that reported prior experience with the PDSA cycle or similar structured process versus the five schools that did not. Below we describe an overall cross-case tendency related to prior structured experience. We then highlight factors that were perceived in a similar way across schools, followed by factors that varied. Lastly, we present some examples of interactions between factors. Alongside these results, we refer to Tables 2, 3, 4 and 5 that contain exemplary quotes (Q1–Q59) to illustrate the valence coding and provide contextual nuance to our key findings.

**Table 2:** Prior Experience with a Structured Approach

School	Structured Approach	#	Quote
A	Present	Q41	*“However, our overall approach to implementation is already very structured…I'll put it this way, it's constantly growing. It goes on and on.”* (School A: 31)
B	Present	Q42	*“So it's already very structured, institutionalized, where I've seen that if you just do it regularly, then it just becomes second nature.”* (School B: 20)
C	Present	Q43	*"We are, and the school leadership or the deputy principal is already very structured. And when we develop something, like a media concept, it runs very smoothly."* (School C: 173*)*
D	Present	Q44	*“We're actually already quite far along. We recognized things and then tried to land some projects. Also with people from outside. To implement them, to evaluate them. But of course, the harvest, we're not quite there yet, it just takes time.”* (School D: 124)
E	Absent	Q45	*"I actually don’t see any structure in the processes yet."* (School E: 53)
F	Absent	Q46	*“It is not yet anchored in any structure”* (School F: 20) *“These are always just small, selective things that come up from time to time, exactly, but they don't, as they say, keep repeating themselves in a rotating loop.”* (School F: 49)
G	Absent	Q47	*“I just think it's all a bit piecemeal, not structured”* (School G: 21) *“What we lack is evaluation. There's nothing there yet, because the other things are also a bit behind.”* (School G: 32)
H	Absent	Q48	*We do a lot, but not with such a background and structured approach.* (School H: 4)
I	Absent	Q49	*Again, there are so many individual aspects that count, but still no single common thread, no structure.* (School I: 55)

Quotes from the interviews

**Table 3:** Commonalities Across Schools

Domain	Factor	Valence	#	Quote
Individuals	Parent Engagement	Barrier	Q1	*"Parents are the danger, it's not a nice thing the way they set the example for their children."* (School G: 28)
			*Q2*	*“So you definitely have to ask the parents, yes, you can, you can teach up to a certain point, but with the parent at home, as I said, just throwing the pizza in the oven and turning on the TV. […] By then, everything we said at school is passé, to put it bluntly. […] That's a very high goal, where you always get them on board and say “Guys, we can only initiate things and you have to practically end them”.” (School F: 84)*
	Principals	Facilitator	*Q3*	*“And that's why I'm so glad, that you [the principal] got this going. It's really great. Not every school does this, some just continue floating in their own comfort zone.” (School F: 128)*
			*Q4*	*As a school principal, I now also have the obligation to promote health (School B: 97)*
	Steering Group	Facilitator	*Q5*	*I would perhaps see it as a strength that the first step has basically been taken, that the school is prepared to engage with this topic at all. That there are teachers and parents who support this topic, i.e. that they have really opened up to tackling it. I definitely see that as an encouragement, because it's not as if someone came in from outside and said: “Well, you have to implement this now”. In a way, that was the intrinsic motivation on the part of the school. (School F: 98)*
			*Q6*	*I simply said that it would be a great fit and then asked around who else would like to take part and then one of my colleagues and the school management immediately agreed and then we said we'd just do it. (School I: 16)*
Inner Setting	Tension for Change	Facilitator	*Q8*	*“And I think, in general, these 4 priorities of the program are very important for us, where I have to say that we also have to work on them and not always just on the curriculum.”* (School E: 49)
			*Q9*	*“But I think that now that it's all on the increase, […] I think more needs to be done in this area. I think we need to do more in this area.”* (School C: 147)
	Compatibility	Facilitator	Q10	*“And we haven't had that at the school yet and we now have to draw up a protection concept and it [the health promotion intervention] fits in really well.”* (School I: 25)
	Materials & Equipment	Facilitator	Q11	*“Yes, it has to be said that we are also well equipped technically.”* (School F: 20)
			Q12	*“We also have a lot of play materials […] and now we also have the program boxes”* (School G: 44–46)
	Communication	Facilitator	Q13	*“So it's also really short distances and you don't have to make an appointment with the boss to start a program, he just sits next to me and says yes. But I really think that makes things a lot easier* *So the communication here in the team is easy and that's really great.” (School D: 165–166)*
			Q14	*“As far as evaluation is concerned, we are actually in a good position. We have our own school app and we use it to carry out regular evaluations.” (School A: 39)*
	Mission Alignment & Culture	Facilitator	*Q15*	*“We have already won two awards […]. And we now want to put the whole thing together into a complete package […]. And that's actually how we came to say that this is actually the right puzzle to complete the picture.” (*School B: 9)

Exemplary quotes from the interviews

**Table 4:** Differences Across Schools

Domain	Factor	Valence	#	Quote
Individuals	Teacher Engagement	Barrier	Q16	*"The colleagues' skepticism is a barrier."* (School E: 70)
		Mixed	Q17	*“It always depends, so some colleagues really do a lot […] and in other classes it's less. Very different.” (*School H: 16*)*
		Facilitator	Q28	*"And also the staff [is a facilitator] —since we are all people who chose this school because of these factors [the school mission and health focus], everyone here is genuinely committed to it." (*School B: 90)
Inner Setting	Time & Relative Priority	Barrier	Q19	*"These programs are well designed but simply too much alongside regular school tasks."* (School D: 150)
			Q20	*"The main thing that makes it really complicated is simply the time."* (School E: 97)
			Q21	*“So you can expand the topic endlessly, yes. But you just have to see how far, what you do. Do you take something away from the lessons and that's the thing.” (*School F: 60*)*
		Facilitator	Q22	*“Strictly speaking, you have to say that it's all there in the curriculum and you just have to be clever and realize that it's part of it.”* (School B: 78)
	Work Infrastructure	Barrier	Q23	*"Since we are not a full-day school, they are picked up at 1 PM, which means we don't have the option to say that we will extend activities. We really try to do this through support courses and other programs… However, much more could be done."* (School H: 31)
			Q24	*"The shortage of staff is really an issue right now, with the wave of illness and the lack of teachers […] and it’s not just affecting us but also other schools. (*School B: 92*)*
		Mixed	Q25	*"Teachers pretty well, but as I said, […] everything that is important, you need additionally.” (*School D: 179)
		Facilitator	Q26	*"I mean, we are a full-day school, and that naturally brings a different perspective on the child, where you can see that we spend a long time together here. We are finding a way to structure the lessons so that it creates a health-promoting daily routine for everyone involved." (*School B: 13)
			Q27	*"We have a new school psychologist starting today who will really provide support." (*School F: 36)
	Space	Barrier	Q28	*“Space is a big issue, big space problem. Has been an issue for over 8 years” (*School I: 65)
			Q29	*“Well, that the school gym is not always available. […] Yes, so the spatial conditions, that we really only ever have the classrooms or the schoolyard available and the gym is not always free” (*School E: 72)
		Facilitator	Q30	*“Yes, how are you set up there (in terms of space)?” W: “Hey great, I think it's great. […] It's mega here.” (*School D: 154–158)
			Q31	*“We are actually very well equipped in terms of space.”* (School B: 21)
Outer Setting	Local Conditions	Barrier	Q32	*“The gyms are completely packed, it has to be said. This goes for other schools and clubs as well. Everything is full, which is nice, of course. But in the afternoons, for example, there are hardly any sports clubs because there’s no space. The people are available, but not the venue." (*School I: 84–85)
			Q33	*“The schoolyard is also rented by the city and the question is who does what […] and it's a traffic training area, so it's also a parking lot for people who go to the sports hall, so it's not separated.” KS: Ok, and they said they weren't allowed to change that either. “Yes, so most of the schoolyard has to stay like this because of the traffic signs.” (*School H: 48–50)
		Facilitator	Q34	*"We have fields and forests here—really ideal conditions." (*School G: 30)
	Partnerships	Barrier	Q35	*“Even with the cafeteria, which opens during the 2nd break. They only sell crap.” (*School I: 45*)*
		Facilitator	Q36	*"We have many after-school sports groups, and we also receive some support from associations, or we have partnerships with sports clubs." (*School F: 65)
			Q37	*"Nutrition is such that we are in close contact with the food providers; we are definitely working to promote health and collaborate closely." (*School B: 53)
	Financing	Barrier	Q38	*“Financial resources, that's always somewhat of a problem (*School I: 79*)*
			Q39	*We can, but then the question is who finances it (…). We wanted a multifunctional space like this upstairs, but it costs €20,000 and then it's always rejected.” (*School H: 50)
		Facilitator	Q40	*“So we can actually add financial resources […] that's not the problem here. So there's also something from the school board, from the side, so if I have a budget for next year, I can definitely take something from that.” (*School B: 103)

Exemplary quotes from the interviews

**Table 5:** Interactions of Factors

Domain	Factor	Valence	#	Quote
Outer Setting	Financing & Local Conditions → Partnerships & Connections	Barrier	Q50	*“Our caterer is a disaster, a real disaster. That would be great, of course, but it's not going to happen, you can't get rid of them. […] Yes, and because there aren't any around here who do that. And it's also a cost factor.* (School I: 31–33)
Outer Setting – Inner Setting	Financing → Space	Barrier	Q51	*“The problem at the moment is that we don't have a school kitchen. But we're doing a charity run soon, where we want to raise money for it. Because we can only tackle the issue of nutrition if we have a functioning school kitchen where we can try things out.”* (School F: 49)
Outer Setting – Inner Setting	Financing → Work Infrastructure (Staff Level)	Barrier	Q52	*“So if we have to get finances from external sources, then it becomes difficult, so as soon as it's somehow done through the school, then it's not a problem, but as soon as it's something bigger and people have to be hired, we reach our limits.”* (School D: 175)
Inner Setting – Individuals Domain	Staff Level, Time → Relational Communications → Parent Engagement	Barriers	Q53	*“Parents should be brought on board much more. I think this is neglected due to lack of patience, lack of time and the many sick leaves. More conversations should be held if capacity allows.”* (School I: 80–83*)*
Inner Setting – Individuals Domain	Mission Alignment/Culture → Teacher Engagement	Facilitator	Q54	*“And also the staff, because we are all people who chose this school because of the factors, and they are all people here who want it too.”* (School B: 90)
			Q55	*“So there will be colleagues who say they don't feel like it […] But I would say that 80% of colleagues would already be open to it. We are also a committed school here.”* (School C: 317)
Inner Setting – Individuals Domain	Mission Alignment/Culture → Parent Engagement	Facilitator	Q56	*“The fact that they chose this school because of the concept is, of course, another driving force, […] of course, we want it out of our own conviction, but the parents are also behind it and want to support it.”* (School B: 15)
Inner Setting – Individuals Domain	Relational Communications → Teacher and Parent Engagement	Facilitator	Q57	*“Communication was transparent, it was also announced and presented to the entire school management. Also in the committee: that is the school management, certain teachers, educational specialists, secretaries. And they then take the information back to their areas. Since the program is also suitable for substitute teaching, the program was then briefly presented to the entire teaching staff at the general conference.”* (School A: 12)
			Q58	*“Otherwise it would be nice if there was more parental involvement and more interest. Among parents and teachers. But we are confident about that too. We have laid the groundwork, we have communicated it […]”* (School A: 14)
Inner Setting – Individuals Domain	Relational Communications → Work Infrastructure	Facilitator	Q59	*“We have established communication structures where we exchange information. I wanted to get away from the questionnaires, evaluating, extra work. This is how it fits into the teaching context, the daily work routine. We now have weekly situations where we exchange ideas and get feedback.* (School A: 40–41)

Exemplary quotes from the interviews

### Cross-case overview: role of prior structured experience

Across the nine schools, responses to the question about prior experience with the PDSA cycle or a similar structured process fell into two clear categories (Table 2, Q41–Q49). Some representatives emphasized working in a structured manner and having prior experience (e.g., “We are, and the school leadership or the deputy principal is already very structured. And when we develop something, like a media concept, it runs very smoothly” – School C), while others reported unstructured approaches and a lack of experience (e.g., “I actually don’t see any structure in the processes yet” – School E). None of the schools indicated having fully applied the PDSA cycle before receiving program training.

From these findings, a notable cross-case tendency emerged: schools that described prior structured experience also anticipated a higher proportion of implementation facilitators and fewer barriers, while those without such experience reported the opposite. This distinction was not linked to sampling factors (urban/rural location, socio-economic index, federal state) but rather to whether schools had engaged in structured approaches in the past.

#### Commonalities across schools

As expected, several well-known determinants also appeared across all schools. *Parent Engagement* was reported as an important barrier by all schools. The school representatives felt like they cannot achieve as much when parents are not involved or even act counterproductively to the health promotion efforts (Table 3 – Q1). Bringing the parents on board was a metaphor frequently used, however doing so was challenging for all schools (Table 3 – Q2).

Conversely, *Principal* support and engagement from *Steering Group* members were reported as anticipated facilitators across all schools (Table 3 – Q6). Other commonly reported facilitators included a general *Tension for Change*, *Compatibility* of program components with existing HPA, and the availability of sufficient *Materials & Equipment* (Table 3 – Q8–Q12).

Two further Inner Setting factors were exclusively reported as facilitators, though they were only mentioned by about half of the schools. *Relational Communications* referred to communication structures within the school that were expected to support the implementation of HPA through the PDSA cycle (Table 3 – Q13, Q14). *These accounts came exclusively from schools with prior structured experience. Mission Alignment & Culture* reflected the extent to which PDSA cycle utilization for HPA implementation was aligned with the school’s broader mission and culture (Table 3 – Q15).

#### Differences across schools

*Teacher Engagement* was perceived as mixed (i.e., barrier and facilitator) by most schools because some but not all teachers were reported to be supportive (Table 4 – Q17). However, some schools reported having committed teachers only (Table 4 – Q18), while others struggle with stronger skepticism among their group of colleagues (Table 4 – Q16).

*Relative Priority* and *Time* were cited by almost all schools as a barrier, with one exception. Competing curricular demands were frequently mentioned as limiting factors (Table 4 – Q19–Q21). One school, however, emphasized that integrating HPA into the curriculum could mitigate this issue (Table 4 – Q22).

*Work Infrastructure* comprised statements regarding both the structural organization (e.g., whether the school was a full-day school) and staff levels. Some schools felt that not being a full-day school limited opportunities for implementing HPA (Table 4 – Q23), whereas others saw being a full-day school as a facilitator for integration into daily routines (Table 4 – Q26). Regarding staff, some schools reported having enough teachers relative to student numbers but insufficient support personnel, resulting in a mixed rating (Table 4 – Q25). Others had additional staff such as school psychologists or social workers and therefore perceived staffing as a facilitator (Table 4 – Q27). Schools with insufficient teachers and lacking other personnel cited staffing as a barrier (Table 4 – Q24).

*Space*, referring to physical infrastructure, was mentioned by all schools but assessed very differently: some perceived their facilities as a barrier (Table 4 – Q28, Q29), while others described them as a facilitator (Table 4 – Q30, Q31).

*Local Conditions*, part of the Outer Setting domain, were consolidated with *Policies & Laws*, which were mentioned only once. Examples ranged from supportive environments such as nearby forests (facilitator; Table 4 – Q34) to limited outdoor space (Table 4 – Q32) or even a schoolyard used as a public parking lot (barrier; Table 4 – Q33).

*Partnerships and Connections* with external organizations such as sports clubs were another factor with mixed valence. While most schools reported supportive partnerships (Table 4 – Q36, Q37), some described difficulties that turned partnerships into barriers (Table 4 – Q35).

Finally,* Financing* was mentioned in all schools but with divergent interpretations. Some representatives emphasized lack of financial resources as a fundamental barrier (Table 4 – Q38, Q39), while others reported sufficient funding and thus saw financing as a facilitator (Table 4 – Q40). Interestingly, none of the schools that reported prior structured experience described financing as a barrier, whereas this concern was more common among those without such experience.

### Interactions between factors

Several schools described instances where one contextual factor appeared to influence or reinforce another. These accounts varied and were not consistent across cases, but they illustrate how factors were sometimes experienced in combination. *Financing* was described as shaping other determinants. A lack of resources was mentioned as constraining *Partnerships & Connections*, limiting the use of available *Spac*e, and affecting *Work Infrastructure* (School I, F, D; Table 5 – Q50–Q52). In one case, low staff levels (part of *Work Infrastructure*) combined with low *Relative Priority & Time* were said to *weaken Relational Communications* and ultimately *Parent Engagement* (School I; Table 5 – Q53). Positive reinforcing dynamics were also reported. In some schools, a strong *Mission Alignment* and health-promoting *Culture* were perceived as supporting *Teacher and Parent Engagement* (School B, C; Table 5 – Q54–Q56). Established *Relational Communications* were further described as enabling *Teacher and Parent Engagement* as well as *Work Infrastructure* (School A; Table 5 – Q57–Q59).

## Discussion

This paper aimed to identify key contextual factors perceived to facilitate or hinder the future utilization of the PDSA cycle for implementing HPA, to examine which factors differ across schools, and to explore whether such differences reveal notable tendencies across cases. As an extension of the framework method, our MHM facilitated the identification of cross-case tendencies, whereby schools with prior experience in a structured process similar to the PDSA cycle reported more contextual facilitators to implementing HPA (e.g., communication structures), whereas those without such experience reported more barriers (e.g., financial constraints).

Although the MHM allowed visual comparisons and uncovered this tendency, it raises the broader question of causality: does experience with structured processes help schools overcome contextual barriers, or are schools with fewer barriers simply better positioned to work in a structured way? Alternatively, prior experience and the number of perceived barriers versus facilitators might both have another underlying cause such as variability in leadership style, which is known to be of great importance for change processes in schools [46–49]. In our study, however, supportive leadership was reported as given across almost all schools. Instead, communication structures emerged as a facilitator only in schools with prior experience, while financial barriers were reported primarily by schools without such experience, potentially pointing to these as important factors to consider as we continue to study the extent to which schools use PDSA to implement HPA programs. However, since our design did not allow us to establish causal ordering, these findings should be understood as exploratory and hypothesis-generating.

At the same time, the findings may still have practical implications for improving schools’ capacity to apply the PDSA cycle. Consistent with school improvement research highlighting the importance of prior experience for adopting the PDSA cycle [26], screening schools for prior experience and tailoring implementation strategies accordingly could be considered a starting point for moving beyond “one-size-fits-all” approaches [28]. Schools with little prior experience may benefit from more intensive support to adopt and apply the PDSA cycle or to alter other contextual factors that may serve as barriers, thereby building organizational capacity, whereas schools with more prior experience may require less intensive support.

Independent of prior structured experience, several barriers and facilitators emerged that appear broadly relevant across schools. Parent engagement was universally described as a challenge, echoing extensive evidence that parents play a pivotal role in school health promotion yet are often difficult to involve [27, 47, 50, 51]. Work infrastructure, in terms of staffing levels was a recurring barrier that is consistent with the systemic issue of the Germany-wide teacher shortage [52], whereas on organizational structure level, operating as a full-day school was perceived as a consistent facilitator. Finally, competing curricular demands and lack of time were frequently mentioned across all schools, aligning with prior studies that show that health promotion is often experienced as competing with schools’ primary task of providing foundational education [50, 53, 54].

Further, our findings show that contextual factors were not only perceived in isolation but also in interaction, reinforcing one another in unique ways. For example, a lack of financial resources was described as constraining partnerships, limiting the use of available space, and affecting staffing. In another case, staff shortages combined with competing curricular demands were said to weaken communication structures and ultimately hinder parent engagement. Positive links were also mentioned: strong mission alignment was perceived as fostering teacher and parent engagement, and established communication structures were seen as enabling collaboration across different areas, including staffing and parental involvement. These dynamic interactions are not readily apparent in the MHM but illustrate the value of qualitative workshops in capturing how contextual factors may influence each other in complex ways that are unlikely to emerge from survey-based assessments or purely deductive frameworks. While the same dynamics were not reported in all schools, they point to potentially transferable mechanisms (e.g., root causes versus symptoms) that merit further investigation. In this sense, the findings are hypothesis-generating and contribute to the identification of core contextual factors that may shape or be shaped by a school’s capacity to utilize structured change processes such as the PDSA cycle.

Beyond these findings, our study also offers a methodological contribution by showing how a combination of the framework method with a visual extension can support cross-case comparison. While the framework method remained the primary analytic approach [34]*, *extending it with a color-coded MHM provided a transparent way to synthesize valence ratings and to compare contextual conditions across multiple schools. This visualization did not replace in-depth qualitative interpretation but complemented it by highlighting tendencies and facilitating structured comparison. In this way, the approach proved valuable for capturing both well-established key contextual barriers and more nuanced tendencies, thereby strengthening confidence in its transferability to other multi-site implementation studies.

This study has several strengths and limitations that should be considered. A key strength is the detailed reporting of our methodological approach. We believe that this adds rigor to our findings and enhances transferability of the approach to other implementation research projects, where contextual conditions are expected to be heterogeneous. Regarding results, the approach was exploratory and revealed some novelties as well as reproduced well-known results in the field. However, it necessitates future research to generate more robust findings, as the sampling strategy and resulting small sample size limit the generalizability of the results. Additionally, all participating schools had already decided to adopt and implement the program. As Morgan et al. emphasize, adoption decisions in schools are often hindered by barriers such as limited leadership support or low staff motivation [55]. Such barriers did not prominently appear in our data, which likely reflects the higher baseline motivation of our sample schools. At the same time, discussing leadership and staff motivation in the presence of their members may have influenced responses due to social desirability bias*.* Finally, although the combined framework and MHM approach enhanced transparency and comparability across schools, the very open and explorative nature of the study led to gaps in the data, leaving some aspects underexplored. More targeted follow-up questions to schools could have provided deeper insights into the identified barriers and facilitators.

Future research could extend our hypothesis-generating findings with complementary methodological approaches. Participatory causal loop diagramming could be applied during workshops to make perceived causal chains and feedback loops more explicit and to co-create visual representations of reinforcing or mitigating dynamics with participants [56]. At a larger scale, configurational comparative methods such as Coincidence Analysis (CNA) are suitable for identifying combinations of factors that are sufficient and necessary for successful PDSA utilization for HPA implementation [57]. Because CNA requires a preselection of factors, our study provides a useful starting point by narrowing down potential difference-makers that can be carried forward into such analyses [33].

## Conclusion

This exploratory study examined contextual factors that shape schools’ perception of the implementability of PDSA cycles for health promotion implementation. Across nine schools, we found a tendency for those with prior structured experience to anticipate more facilitators and fewer barriers, while schools without such experience reported more obstacles to HPA implementation. Although causality cannot be inferred, these findings suggest that prior structured experience could be an important consideration for tailoring implementation strategies. Schools with little prior experience may benefit from more intensive support to build organizational capacity and to address contextual factors that systematically differentiated these schools from those with greater experience using structured processes similar to PDSA cycles. Not only did our study provide insights into core contextual conditions under which structured processes have been and may be used, we also illustrated a methodological approach that can be transferred to other multi-site implementation studies to explore and compare heterogeneous contexts. For the Health Promoting School approach, the contextual heterogeneity we found highlights that capacity building may not be a one-size-fits-all endeavor. Structured processes such as the PDSA cycle may provide valuable scaffolding, but their application will likely depend on and/or influence contextual factors.

## Supplementary Information

Additional file 1.

Additional file 2.

Additional file 3.

Additional file 4.

## Data Availability

The pseudonymized interview data used and analyzed during the current study are available from the corresponding author on reasonable request.

## Abbreviations

CFIR: Consolidated Framework for Implementation Research
MHM: Matrix Heat Map
HPA: Health-promoting activities
PDSA cycle: Plan-Do-Study-Act cycle
WHO: World Health Organization
