# Supplementary material for: Visualizing contextual determinants in and across heterogeneous settings: a qualitative study on structured school health promotion implementation
Source: Implement Sci Commun. 2026 Jan 16;7:13. doi: 10.1186/s43058-026-00861-x (PMC12836875; doi:10.1186/s43058-026-00861-x)
Supplement: Supplementary file 1 — Additional file 1. [file 43058_2026_861_MOESM1_ESM.docx]

**School recruitment for the program**

Across three cohorts, approximately 1,370 primary schools were recruited, around 9% of all German primary schools (1), which exceeds common sampling percentages used at the acquisition stage for designing representative studies (2). To ensure representativeness and equity, recruitment was designed to achieve a balanced inclusion of schools with and without prior health promotion experience, schools from both urban and rural areas (3) and approximately 25% of schools from socially disadvantaged contexts (4). Urban–rural classification was based on the population size of the municipalities in which schools were located (5). To identify socially disadvantaged schools, we used **SINUS-Milieus data** (6) as a proxy for local socioeconomic conditions. Specifically, the concentration of the precarious milieu within a 1 km radius of each school was calculated and normalized against regional averages to account for structural demographic differences (7).

**References**

1. Statista. Anzahl der Grundschulen in Deutschland in den Schuljahren von 2010/2011 bis 2023/2024; 2025 [cited 2025 Aug 7]. Available from: URL: https://de.statista.com/statistik/daten/studie/235833/umfrage/grundschulen-in-deutschland/.
2. PIRLS - International Requirements for Sampling, Data Collection, and Response Rates; 2021 [cited 2025 Aug 7]. Available from: URL: https://nces.ed.gov/surveys/pirls/pirls2016/technotes_intlrequirements.asp?utm_source=chatgpt.com.
3. Zehetner E, Janschitz G, Fernandez K. Bildung zwischen Stadt und Land. Der Mythos Stadt-Land im Spiegel aktueller Forschungsbefunde. Z f Bildungsforsch 2022; 12(1):165–77.
4. Weishaupt H. Wann sind Grundschulen in „sozial schwierigen Lagen“ und was bedeutet dies für Lehrkräfte, Schülerinnen und Schüler? DDS 2022; 2022(01):89–111.
5. Bundesinstitut für Bau-, Stadt-, und Raumforschung. Referenztabellen zur Raumgliederung des BBSR; 2023 [cited 2025 Oct 1]. Available from: URL: https://www.bbsr.bund.de/BBSR/DE/forschung/raumbeobachtung/Raumabgrenzungen/downloads/download-referenzen.html.
6. Barth B, Flaig BB, Schäuble N, Tautscher M. Praxis der Sinus-Milieus®. Wiesbaden: Springer Fachmedien Wiesbaden; 2023.
7. Sachweh P, Schröder T, Speer A, Groh-Samberg O. Soziale Milieus. In: Böhnke P, Konietzka D, editors. Handbuch Sozialstrukturanalyse. Wiesbaden: Springer Fachmedien Wiesbaden; 2025. p. 1–24.
