# Supplementary material for: Visualizing contextual determinants in and across heterogeneous settings: a qualitative study on structured school health promotion implementation
Source: Implement Sci Commun. 2026 Jan 16;7:13. doi: 10.1186/s43058-026-00861-x (PMC12836875; doi:10.1186/s43058-026-00861-x)
Supplement: Supplementary file 3 — Additional file 3. [file 43058_2026_861_MOESM3_ESM.docx]

| **Phase** | **Timing** | **Content** | **Notes** | **Materials** |
| --- | --- | --- | --- | --- |
| Introduction | 5 min | Round of introductions, including each participant's role in the school. | Goal: Get to know each other. | None |
| Agenda Presentation | 2 min | What is today's focus? Us taking the learner perspective, participants as experts. | Explain the use of the audio recorder and ensure participants feel comfortable. | None |
| Timeline Exercise | 10 min | Display of a timeline from 0–10 to visualize the upcoming two years. Participants mark their status-quo and define their vision for becoming a HPS (10 on the scale). | Helps identify current status and sets a common goal | Paper timeline, sticky notes, pens |
| Mapping Implementation/Implement-ability | 30 min | Reflect on the state of school health promotion prior to fit4future & determine prior experience with PDSA-cycle-like processes. *“As you know, the main component of the program is learning the Plan-Do-Study-Act (PDSA) cycle, meaning a structured approach to implementing health-promoting activities. How would you assess your previous experience in this regard? How structured has your approach been so far when implementing health promotion activities, for example?”*  Discuss future implementation (implementability) 🡪 When you think about utilizing the PDSA cycle to implement health-promoting activities in the future, what are perceived barriers and facilitators? | Helps understand current status, the school’s approach and facilitates identifying barriers & facilitators | Poster, sticky notes, pens. |
| Open Discussion | 10 min | Reflection and open exchange of additional thoughts or unresolved issues. | Provides closure and addresses all participant concerns. | None |
| Closing | 3 min | Thank participants and outline how the findings will be used for research and practice. | Ensure gratitude is expressed. | None |

**Workshop schedule**
