# Supplementary material for: Visualizing contextual determinants in and across heterogeneous settings: a qualitative study on structured school health promotion implementation
Source: Implement Sci Commun. 2026 Jan 16;7:13. doi: 10.1186/s43058-026-00861-x (PMC12836875; doi:10.1186/s43058-026-00861-x)
Supplement: Supplementary file 4 — Additional file 4. [file 43058_2026_861_MOESM4_ESM.docx]

**CFIR codebook adapted for the use in the school setting**

Aim: identify how contextual factors differ across and co-occur within schools regarding the future application of the PDSA-cycle for HPA implementation

**Adaptation Steps**

Implementation Process Domain:

- Excluded
- Important but beyond the scope of this paper

Innovation Domain:

- Excluded (not necessarily a contextual factor according to literature)
- Not relevant for the research question

Individuals Domain:

- Definition of school-specific roles/stakeholders
- Steering group
- Principals/Leaders
- Teachers as Innovation Deliverers
- Parents as Innovation Supporters
- (children not included, because the goal is on the organizational level)

Outer Setting:

- Almost no changes made
- Local attitudes
- Local conditions (includes policies & laws because of the federal structure of the education system in Germany)
- Partnerships & connections
- Financing
- External pressure

Inner Setting:

- Most changes
- No distinction between structural characteristics and available resources made 🡪 makes no sense in this case
- New categories are:
  - Space
  - Materials & equipment (includes information technology)
  - Work infrastructure includes Staff Levels
  - Relational connections & communications
  - Mission alignment & culture (together)
  - Compatibility
  - Relative priority & Time (inductively created)

| 1. **Outer Setting** | The setting in which the Inner Setting exists, e.g. school district, state.  Project Outer Settings: the community, city, state, the school authorities |
| --- | --- |
|  | The degree to which… |
| 1. Local Attitudes | Definition: Sociocultural values (e.g., shared responsibility in helping recipients) and beliefs (e.g., convictions about the worthiness of recipients) encourage the Outer Setting to support implementation and/or delivery of the innovation. |
| 1. Local Conditions including Policies & Laws | Definition: Economic, environmental, political, and/or technological conditions enable the Outer Setting to support implementation and/or delivery of the innovation.  Example: Local gym halls/swimming pools/forests etc.  Definition: Legislation, regulations, professional group guidelines and recommendations, or accreditation standards support implementation and/or delivery of the innovation  Example: the regulation of how public space close to the school can be used and/or changed to support the implementation e.g. a school yard that is also a parking lot |
| 1. Partnerships & Connections | Definition: The Inner Setting is networked with external entities, including referral networks, academic affiliations, and professional organization networks.  Examples: local sports clubs, catering services |
| 1. Financing | Definition: Funding from external entities (e.g., grants, reimbursement) is available to implement and/or deliver the innovation  Example: receiving enough funding from school authorities |
| 1. External Pressure | Definition: External pressures drive implementation and/or delivery of the innovation. Note: Use this construct to capture themes related to External Pressures that are not included in the subconstructs below.  Includes societal, market and performance measurement pressure. |
| 1. **Inner Setting** | The setting in which the innovation is implemented. The school.    Project Inner Setting: in our case, the inner setting is the school itself as a whole. |
|  | The degree to which… |
| Physical Characteristics & Resources | Definition: Infrastructure components support functional performance of the Inner Setting. Note: Use this construct to capture themes related to Structural Characteristics that are not included in the subconstructs below |
| 1. Space | Layout and configuration of space support functional performance of the Inner Setting.  Example: the building itself – basic materials for the daily business, enough classrooms etc.  Physical space is available to implement and deliver the innovation.  Example: Enough space for physical activity, relax-time, outdoor play, etc. |
| 1. Materials & Equipment together with Information Technology Infrastructure | Supplies are available to implement and deliver the innovation.  Example: Materials for physical activity  Technological systems for tele-communication, electronic documentation, and data storage, management, reporting, and analysis support functional performance of the Inner Setting.  e.g. Ipads in classrooms |
| Organizational & Relational Characteristics & Resources | |
| 1. Work Infrastructure | Organization of tasks and responsibilities within and between individuals and teams, and general staffing levels, support functional performance of the Inner Setting.  Example: lack of staff –being understaffed; the school is organized as a full-day school |
| 1. Relational Connections & Communications | Definition: There are high quality formal and informal relationships, networks, and teams within and across Inner Setting boundaries (e.g., structural, professional).  There are high quality formal and informal information sharing practices within and across Inner Setting boundaries (e.g., structural, professional).  Example: the school uses an app for regular feedback processes |
| Cultural Characteristics |  |
| 1. Culture | Definition: There are shared values, beliefs, and norms across the Inner Setting. Note: Use this construct to capture themes related to Culture that are not included in the subconstructs below.  Example: something that unites the team |
| 1. Mission Alignment | Definition: Implementing and delivering the innovation is in line with the overarching commitment, purpose, or goals in the Inner Setting.  Example: school as a sport school |
| Innovation-specific Characteristics & Resources | |
| 1. Tension for Change | Definition: The current situation is intolerable and needs to change.  Example: Realizing the health behavior/health of the children is suboptimal |
| 1. Compatibility | Definition: The innovation fits with workflows, systems, and processes.  Example: the innovation components fit well with already existing health promotion measures |
| 1. Relative Priority & Time | Definition: Implementing and delivering the innovation is important compared to other initiatives.  Example: other initiatives are at place or need to be taken care of OR a media concept has to be established OR the curriculum has priority  There is time in the daily business to implement and deliver the innovation  Example: Teachers find they simply do not have time to implement the innovation in their daily schedule |
| 1. Access to Knowledge & Information | Definition: Guidance and/or training is accessible to implement and deliver the innovation |
| 1. **Individuals Domain** | The roles and characteristics of individuals. |
| Roles Subdomain | Document the roles applicable to the project and their location in the Inner Setting. |
| 1. Principals | Definition: The school principals. Code if explicitly mentioned, or if it’s clear that COM of the principal is relevant. |
| 1. Steering Group | Definition: Individuals, who are part of the steering group and are thus leading the implementation. |
| 1. Teachers as Innovation Deliverers | Definition: Individuals who are directly or indirectly delivering the innovation |
| 1. Parents as Innovation Supporters | Definition: Parents, of the children visiting the school, who support the delivery of the innovation. |
| 1. Innovation Recipients = Children | Definition: The children, who visit the school and should ultimately profit from the innovation. |
| Characteristics Subdomain | Project Characteristics: [Document the characteristics applicable to the roles in the project based on the COM-B system or role-specific theories. |
|  | The degree to which… |
| 1. Need | The individual(s) has deficits related to survival, well-being, or personal fulfillment, which will be addressed by implementation and/or delivery of the innovation  Example: health deficit on child level |
| 1. Capability | The individual(s) has interpersonal competence, knowledge, and skills to fulfill Role.  Example: teachers’ competency to implementation, parents competency for healthy child-upbringing |
| 1. Opportunity | The individual(s) has availability, scope, and power to fulfill Role  Example: teachers’ opportunities to apply health promoting exercises in class |
| 1. Motivation | The individual(s) is committed to fulfilling Role.  Example: parents’/teachers’/leaders’ motivation to apply the PDSA cycle, implement HPA and support healthy behavior |
